# Impact of persistent PSA after salvage radical prostatectomy: a multicenter study

**DOI:** 10.1038/s41391-023-00728-5

**Published:** 2023-10-06

**Authors:** Felix Preisser, Reha-Baris Incesu, Pawel Rajwa, Marcin Chlosta, Florian Nohe, Mohamed Ahmed, Andre Luis Abreu, Giovanni Cacciamani, Luis Ribeiro, Alexander Kretschmer, Thilo Westhofen, Joseph A. Smith, Thomas Steuber, Giorgio Calleris, Yannic Raskin, Paolo Gontero, Steven Joniau, Rafael Sanchez-Salas, Shahrokh F. Shariat, Inderbir Gill, R. Jeffrey Karnes, Paul Cathcart, Henk Van Der Poel, Giancarlo Marra, Derya Tilki

**Affiliations:** 1 Martini-Klinik Prostate Cancer Center, University Hospital Hamburg Eppendorf, Hamburg, Germany; 2 Department of Urology, University Hospital Frankfurt, Frankfurt, Germany; 3 Department of Urology, Medical University of Vienna, Vienna, Austria; 4 Department of Urology, Medical University of Silesia, Zabrze, Poland; 5 Department of Urology, Mayo Clinic, Rochester, MN USA; 6 Keck Medical Center of USC, USC Institute of Urology, University of Southern California, Los Angeles, CA USA; 7 Urology Centre, Guy’s Hospital, London, UK; 8 Department of Urology, Ludwig-Maximilians University of Munich, Munich, Germany; 9 Department of Urologic Surgery, Vanderbilt University Medical Center, Nashville, TN USA; 10 Department of Surgical Sciences, San Giovanni Battista Hospital and University of Turin, Turin, Italy; 11 Department of Urology, University Hospitals Leuven, Leuven, Belgium; 12 Department of Urology, Institut Mutualiste Montsouris and Université Paris Descartes, Paris, France; 13 Department of Urology, Second Faculty of Medicine, Charles University, Prague, Czech Republic; 14 Hourani Center for Applied Scientific Research, Al-Ahliyya Amman University, Amman, Jordan; 15 Department of Urology, Weill Cornell Medical College, New York, NY USA; 16 Department of Urology, University of Texas Southwestern, Dallas, TX USA; 17 Department of Urology, Netherlands Cancer Institute, Amsterdam, The Netherlands; 18 Department of Urology, University Hospital Hamburg-Eppendorf, Hamburg, Germany; 19 Department of Urology, Koc University Hospital, Istanbul, Turkey

**Keywords:** Prostate cancer, Prostate cancer

## Abstract

**Background and objective:**

Persistent prostatic specific antigen (PSA) represents a poor prognostic factor for recurrence after radical prostatectomy (RP). However, the impact of persistent PSA on oncologic outcomes in patients undergoing salvage RP is unknown. To investigate the impact of persistent PSA after salvage RP on long-term oncologic outcomes.

**Material and methods:**

Patients who underwent salvage RP for recurrent prostate cancer between 2000 and 2021 were identified from twelve high-volume centers. Only patients with available PSA after salvage RP were included. Kaplan-Meier analyses and multivariable Cox regression models were used to test the effect of persistent PSA on biochemical recurrence (BCR), metastasis and any death after salvage RP. Persistent PSA was defined as a PSA-value ≥ 0.1 ng/ml, at first PSA-measurement after salvage RP.

**Results:**

Overall, 580 patients were identified. Of those, 42% (n = 242) harbored persistent PSA. Median follow-up after salvage RP was 38 months, median time to salvage RP was 64 months and median time to first PSA after salvage RP was 2.2 months. At 84 months after salvage RP, BCR-free, metastasis-free, and overall survival was 6.6 vs. 59%, 71 vs. 88% and 77 vs. 94% for patients with persistent vs. undetectable PSA after salvage RP (all p < 0.01). In multivariable Cox models persistent PSA was an independent predictor for BCR (HR: 5.47, p < 0.001) and death (HR: 3.07, p < 0.01).

**Conclusion:**

Persistent PSA is common after salvage RP and represents an independent predictor for worse oncologic outcomes. Patients undergoing salvage RP should be closely monitored after surgery to identify those with persistent PSA.

## Introduction

Salvage radical prostatectomy (SRP) represents one of the treatment options for locally recurrent prostate cancer (PCa) after primary radiotherapy with a curative potential [1]. However, given the rarity of this procedure, the role of prostate-specific antigen (PSA) as a postoperative follow-up marker in the salvage setting is not entirely explored yet [2]. Conversely, PSA is the cornerstone in follow-up after primary radical prostatectomy (RP). The European Association of Urology (EAU) recommends PSA testing after RP, by 6 weeks an undetectable PSA can be expected [3]. A PSA value of ≥0.1 ng/ml within four to eight weeks after RP is defined as persistent PSA [4]. Recently, we reported on the frequency of persistent PSA after primary RP and its impact on long-term oncologic outcomes. Here, persistent PSA was a main predictor for worse oncologic outcomes, namely metastasis-free survival, overall survival (OS) and cancer-specific survival (CSS) [5]. Moreover, a strong association between persistent PSA after RP and biochemical recurrence (BCR) has been reported [6–9]. However, the effect of persistent PSA on oncologic outcomes after SRP is unknown. To address this void, we investigated the association between persistent PSA after SRP and the long-term oncological outcomes, within a multi-institutional center database. Specifically, we focused on the relationship between persistent PSA and BCR after SRP, persistent PSA and development of metastasis, as well as between persistent PSA and OS after SRP. We hypothesized that persistent PSA after SRP represents a predictor for worse oncologic outcomes.

## Material and methods

### Study population

Patients that harbored histology confirmed recurrent prostate cancer after primary therapy, between 2000 and 2021, and had available information on PSA after SRP were identified from a multi-institutional database, derived from twelve high-volume centers. The study was conducted after Institutional Review Board approval and written informed consent was obtained from all patients. Salvage surgery was performed either with an open retropubic or robot-assisted laparoscopic approach as previous described for primary radical prostatectomy [10–12]. Exclusion criteria consisted of metastasis prior SRP (n = 24) and castration resistance at time of SRP (n = 22). These selection criteria yielded 580 patients, who represented the focus of the current study.

### Endpoints

Persistent PSA was defined as a PSA-value ≥ 0.1 ng/ml, at first PSA-measurement after SRP, which was at least 4 weeks after surgery. BCR was defined as two consecutive PSA values ≥ 0.2 ng/ml after SRP. BCR was calculated as the time from SRP to development of biochemical recurrence or last follow-up. Metastasis-free survival (MFS) was defined as positive imaging after development of BCR after SRP. MFS was calculated at the time from SRP to development of metastasis or last follow-up.

Overall survival (OS) was calculated as the time from SRP to death or last follow-up.

### Statistical analyses

Descriptive statistics included frequencies and proportions for categorical variables. Medians and interquartile ranges were reported for continuously coded variables. The Chi-square tested the statistical significance in proportions’ differences. The Mann-Whitney U test examined the statistical significance of medians’ differences, respectively.

Two sets of uni- and multivariable logistic regression models were used to assess the relationship between individual patients characteristics at SRP and the development of persistent PSA. Specifically, the first model tested for preoperative patients characteristics and the second model, respectively, for perioperative characteristics and the development of persistent PSA.

Kaplan-Meier analyses graphically depicted BCR-free, MFS and OS rates. Two sets of univariable and multivariable Cox regression models were fitted to test the relationship between persistent PSA after SRP and the oncologic outcomes. Specifically, the first set of Cox regression models focused on persistent PSA and the development of BCR and the second set of Cox regression models focused on the relationship between persistent PSA and death after SRP. Adjustment was made for the covariates: preoperative PSA value, pathologic tumor stage, surgical margin status, lymph node status, pathologic Gleason Score, who all showed a significant association in univariable analyses with BCR (Supplementary table 1). The models testing for overall survival were additionally adjusted for the Charlson Comorbidity Index (CCI).

R software environment for statistical computing and graphics (version 4.2.2, Vienna, Austria) was used for all statistical analyses. All tests were two sided with a level of significance set at *p* < 0.05.

## Results

### Descriptive statistics and patient predictors for persistent PSA

Of the 580 identified patients, 42% (n = 242) harbored persistent PSA (Table 1). Median follow-up after SRP was 38 months (interquartile range [IQR]: 20–69 months), median time to SRP was 64 months (IQR: 36–114 months) and median time to first PSA after SRP was 2.2 months (IQR: 1.4–4.1 months). 53% and 47% were treated with an robotic and open approach, respectively. Patients with persistent PSA had higher median preoperative PSA values (5.8 vs. 4.4 ng/ml, p < 0.01), higher proportion of positive margins (40 vs. 24%, p < 0.001), more frequently harbored pathologic stage ≥pT3b (46 vs. 24%, p < 0.001) and lymph node invasion (28 vs. 6.4%, p < 0.001).

**Table 1:** Descriptive characteristics of salvage radical prostatectomy patients.

Variable	Persistent PSA, n = 242 (42%)	Undetectable PSA, n = 338 (58%)	p-value
Preoperative PSA, ng/ml, median (IQR)	5.8 (3–9.1)	4.4 (2.8–7.2)	0.01
Age at SRP, yrs, median (IQR)	66 (61–69)	66 (61–71)	0.8
Operating time, minutes, mean (SD)	187 (90)	179 (64)	0.3
Hospital stay, days, median (IQR)	3 (2–7)	3 (1–7)	0.1
Time to first PSA after SRP, months, median (IQR)	2.8 (1.4–4))	2 (1.4–4.2)	0.9
Time to SRP from first PCa diagnosis, months, median (IQR)	63 (37–100)	65 (33–117)	0.9
Number lymph nodes removed, median (IQR)	12 (6–19)	12 (6–18)	0.9
Primary treatment type, n (%)			0.003
External beam radiotherapy	128 (58%)	151 (46%)	
Brachytherapy	49 (22%)	68 (21%)	
Focal therapy	45 (20%)	110 (33%)	
Charlson comorbidity Index, n (%)			0.009
0	153 (63%)	184 (54.4%)	
1	31 (13%)	33 (9.8%)	
>1	58 (24%)	121 (35.8%)	
Nerve sparing, n (%)			<0.001
none	190 (80%)	211 (63.9%)	
unilateral	19 (8.0%)	31 (9.4%)	
bilateral	29 (12%)	88 (26.7%)	
Pathologic tumor stage, n (%)			<0.001
≤pT2	92 (38%)	178 (53%)	
pT3a	39 (16%)	77 (23%)	
≥pT3b	111 (46%)	81 (24%)	
Surgical approach, n (%)			<0.001
Open	144 (60%)	129 (38%)	
Robotic assisted	98 (40%)	209 (62%)	
Biopsy Gleason prior SRP, n (%)			<0.001
≤6	39 (18%)	45 (16%)	
7	92 (42%)	172 (60%)	
≥8	86 (40%)	68 (24%)	
Pathologic SRP Gleason, n (5)			<0.001
≤6	15 (6.5%)	23 (7.4%)	
7	119 (51.3%)	207 (66.3%)	
≥8	98 (42.2%)	82 (26.3%)	
Positive surgical margins, n (%)	97 (40%)	81 (24%)	<0.001
Lymph node status, n (%)			<0.001
pN0	138 (59%)	216 (69.2%)	
pN1	66 (28%)	20 (6.4%)	
pNx	31 (13%)	76 (24.4%)	

*IQR* interquartile range, *SD* standard deviation, *SRP* salvage radical prostatectomy.

In the multivariable logistic regression models (Table 2 - clinical model) testing for preoperative patients characteristics and the development of PSA persistence, higher preoperative PSA (Odds ratio (OR): 1.04, 95%-confidence interval (CI): 1.01–1.08, p = 0.02) and biopsy Gleason 8–10 (OR: 1.81, 95%-CI: 1.14–2.90, p = 0.01) prior SRP were independent predictors for development of PSA persistence after salvage RP. Focal therapy was protective (OR: 0.55, 95%-CI: 0.34–0.89, p = 0.02) for development of PSA persistence, related to external beam radiotherapy as reference.

**Table 2:** Multivariable logistic regression models predicting persistent PSA (≥ 0.1 ng/ml) after salvage radical prostatectomy.

	OR	95%-CI	*P* value
Clinical model			
Age	0.99	0.96–1.02	0.5
Preoperative PSA	1.04	1.01–1.08	0.02
Biopsy Gleason ≤ 6 (referent)	1.00	-	-
Biopsy Gleason 7	1.01	0.60–1.69	0.9
Biopsy Gleason 8–10	1.81	1.14–2.90	0.01
Time to SRP	0.99	0.99–1.01	0.4
Percutaneous radiotherapy (referent)	1.00	-	-
Brachytherapy	0.95	0.58–1.57	0.8
Focal therapy	0.55	0.34–0.89	0.02
Pathological model			
Age	0.98	0.95–1.01	0.1
Preoperative PSA	0.99	0.98–1.02	0.8
Pathologic stage ≤ T2c (referent)	1.00	-	-
Pathologic stage T3a	1.03	0.60–1.75	0.9
Pathologic stage T3b	1.78	1.09–2.89	0.02
Pathologic Gleason ≤ 6 (referent)	1.00	-	-
Pathologic Gleason 7	0.76	0.35–1.66	0.5
Pathologic Gleason 8–10	1.31	0.58–3.02	0.5
Negative surgical margin (referent)	1.00	-	-
Positive surgical margin	2.04	1.32–3.18	<0.01
Pathologic lymph node status N0 (referent)	1.00	-	-
Pathologic lymph node status N1	3.52	1.96–6.51	<0.001
Pathologic lymph node status Nx	0.99	0.57–1.71	0.9
Open SRP (referent)	1.00	-	-
Robotic SRP	0.37	0.24–0.56	<0.001

*OR* Odds Ratio, *CI* Confidence interval, *PSA* prostatic specific antigen, *SRP* salvage radical prostatectomy.

In the multivariable logistic regression models (Table 2 – pathologic model) testing for perioperative patients characteristics and the development of PSA persistence, lymph node invasion (OR: 3.52, 95%-CI:1.96–6.51, p < 0.001), pathologic stage ≥T3b (OR: 1.78, 95%-CI: 1.09–2.89, p = 0.02) and positive surgical margins (OR: 2.04, 95%-CI: 1.32–3.18, p < 0.01) were all independent predictors for development of PSA persistence after SRP. Conversely, patients treated with robotic SRP had a lower risk for persistent PSA (OR: 0.37, 95%-CI: 0.24–0.56, p < 0.001).

### Persistent PSA and biochemical recurrence

**Fig. 1:**
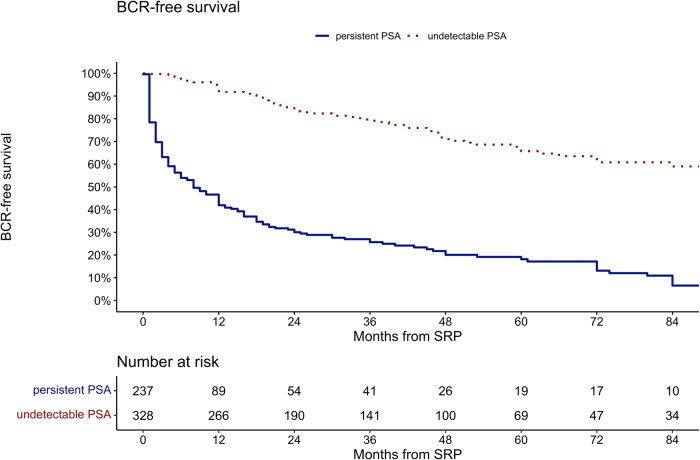
Kaplan Meier curves depicting BCR-free survival after SRP. Patients stratified according to undetectable PSA (red dotted line) and persistent PSA (blue line; log-rank test, p < 0.001).

Median BCR-free survival (Fig. 1) was 8 months vs. not reached (p < 0.001) for patients with persistent vs. undetectable PSA after SRP time. At 84 months after SRP, BCR-free survival was 6.6 vs. 59%, respectively. For the entire cohort, median BCR-free survival was 48 months and at 84 months after SRP BCR-free survival was 34%.

Additionally, in multivariable Cox models, persistent PSA (Table 3) was an independent predictor for development of BCR (HR: 5.47, 95%-CI: 3.98–7.51, p < 0.001), after adjusting for preoperative PSA value, pathologic tumor stage, surgical margin status, lymph node status and pathologic Gleason Score.

**Table 3:** Multivariable Cox regression models predicting biochemical recurrence and death after salvage radical prostatectomy.

	Predicting BCR			Predicting death		
	HR	95%-CI	p -value	HR	95%-CI	p -value
Undetectable PSA postoperative	1.00	-	**-**	**-**	**-**	**-**
Persistent PSA postoperative	5.47	3.98–7.51	<0.001	3.07	1.42–6.63	<0.01
Preoperative PSA	1.00	0.99–1.01	0.6	1.01	0.98–1.04	0.6
Pathologic stage ≤ T2c (referent)	1.00	-	-	1.00	-	-
Pathologic stage T3a	1.17	0.79–1.74	0.4	0.58	0.20–1.65	0.3
Pathologic stage ≥ T3b	1.54	1.10–2.15	0.01	1.11	0.53–2.31	0.8
Pathologic Gleason ≤ 6 (referent)	1.00	-	-	1.00	-	-
Pathologic Gleason 7	3.24	1.31–8.04	0.01	0.96	0.31–2.97	0.9
Pathologic Gleason 8–10	4.45	1.77–11.2	<0.01	0.98	0.30–3.27	0.9
Negative surgical margin (referent)	1.00	-	-	1.00	-	-
Positive surgical margin	0.90	0.67–1.21	0.5	0.93	0.46–1.89	0.8
Pathologic lymph node status N0 (referent)	1.00	-	-	1.00	-	-
Pathologic lymph node status N1	1.40	1.01–1.95	0.04	3.14	1.54–6.41	<0.01
Pathologic lymph node status Nx	1.54	1.07–2.22	0.02	1.43	0.57–3.59	0.4
CCI 0 (reference)				1.00	-	-
CCI 1				0.68	0.25–1.82	0.4
CCI ≥ 2				2.34	1.23–4.46	<0.01

*CCI* Charlson comorbidity index, *HR* Hazard Ratio, *CI* Confidence interval, *PSA* prostatic specific antigen.

### Persistent PSA and development of metastasis

**Fig. 2:**
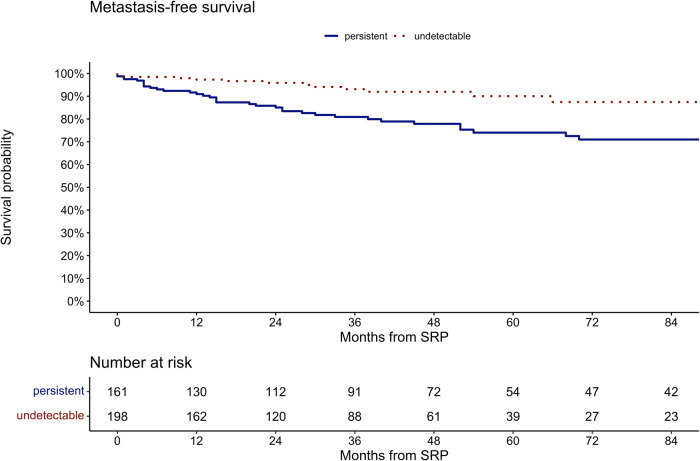
Kaplan Meier curves depicting Metastasis-free survival after SRP. Patients stratified according to undetectable PSA (red dotted line) and persistent PSA (blue line; log-rank test, p < 0.01).

At 84 months after SRP (Fig. 2), MFS was 71 vs. 88% for patients with persistent vs. undetectable PSA after SRP (p < 0.01). For the entire cohort, median MFS was not reached and at 84 months after SRP MFS was 79%. Missing information in the follow-up regarding MFS prevented us from performing multivariable models predicting metastasis.

### Persistent PSA and overall survival

**Fig. 3:**
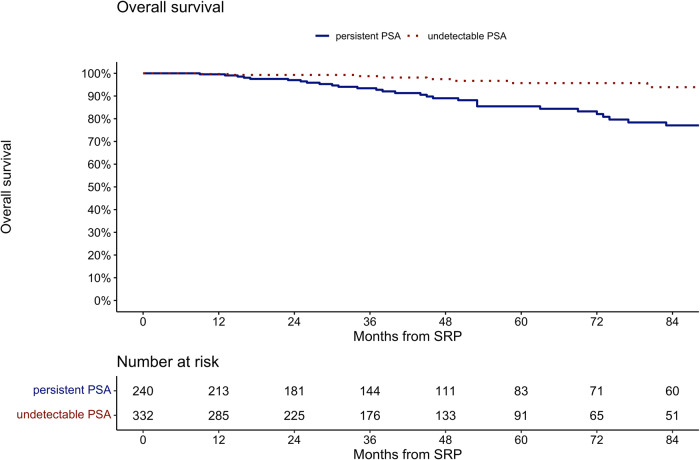
Kaplan Meier curves depicting overall survival after SRP. Patients stratified according to undetectable PSA (red dotted line) and persistent PSA (blue line; log-rank test, p < 0.001).

At 84 months after SRP (Fig. 3), overall survival was 77 vs. 94% for patients with persistent vs. undetectable PSA after SRP (p < 0.001). For the entire cohort, median overall survival was 228 months and at 84 months after SRP overall survival was 85%.

Additionally, in multivariable Cox models, persistent PSA (Table 3) was an independent predictor for death (HR: 3.07, 95%-CI: 1.42–6.63, p < 0.01), after adjusting for preoperative PSA value, pathologic tumor stage, surgical margin status, lymph node status, pathologic Gleason Score and CCI.

## Discussion

PSA testing is the cornerstone in follow-up of patients with PCa after RP. Postoperatively persistent PSA helps to identify patients at risk for worse long-term oncologic outcomes, which might qualify for further treatment options. However, persistent PSA after SRP and its impact on oncological outcomes has not been investigated yet. To address this void, we tested the relationship between persistent PSA after SRP and BCR, as well as between persistent PSA and death after SRP. Our study revealed several noteworthy findings.

First, within a multi-institutional database between 2000 and 2021, we identified 580 patients who underwent SRP for recurrent prostate cancer and postoperative available information on PSA. Our data represents the largest and most contemporary cohort of SRP patients. The second largest population of SRP patients (n = 427; 2004–2016) was identified within the Surveillance, Epidemiology, and End Results (SEER) database [13]. Other reports with smaller and historic cohorts relied on single-institutional data [(n = 55; 2004–2008) [14]; (n = 55; 2007–2012) [1]; (n = 51; 1983–2002) [15]; (n = 100; 1984–2003) [16]; (n = 199; 1967–2000) [17] or the SEER database (n = 364; 1988–2010) [18]. These numbers underline the rarity of SRP. Concerns of higher complication rates of SRP compared to primary RP might explain the generally low case numbers of SRP. In consequence, the use of multi-institutional databases such as the present one is essential to provide generalizable observations for analyses of SRP patients.

Second, 42% of our cohort of SRP patients harbored persistent PSA. To the best of our knowledge, we are the first to report this rate of persistent PSA within an SRP cohort. In consequence, the current results of persistent PSA distribution in SRP patients cannot be directly compared to previous studies. Conversely, in a cohort of 11,604 patients that underwent primary RP, only 8.8% had persistent PSA [5]. Similarly, McDonald et al. reported 12% of patients with persistent PSA after RP, here defined as PSA ≥ 0.2 ng/ml [19]. These lower proportions of persistent PSA in cohorts of primary RP are contrasted by one study that reported 26% PSA persistence [20]. However, this study only included patients with lymph node invasion (LNI) after RP which is known to be a risk factor for persistent PSA [21]. In consequence, an important observation of our study was that persistent PSA is more common after SRP than after RP.

Third, we identified important differences in baseline characteristics between patients with persistent PSA vs. patients with undetectable PSA after SRP. Specifically, patients with persistent PSA had higher median preoperative PSA values (5.8 vs. 4.4 ng/ml, p < 0.01), higher proportion of positive margins (40 vs. 24%, p < 0.001), more frequently harbored seminal vesicle invasion (46 vs. 24%, p < 0.001) and lymph node invasion (28 vs. 6.4%, p < 0.001). In multivariable logistic regression analyses, multiple preoperative and perioperative patients characteristics were identified as risk factors for the development of PSA persistence after SRP. Specifically, higher preoperative PSA (OR: 1.04, p = 0.02) and biopsy Gleason 8–10 (OR: 1.81, p = 0.01) prior SRP were independent predictors for development of PSA persistence after SRP in the clinical model predicting persistent PSA. Moreover, lymph node invasion (OR: 3.52, p < 0.001), pathologic stage ≥T3b (OR: 1.78, p = 0.02) and positive surgical margins (OR: 2.04, p < 0.01) were independent predictors for development of PSA persistence after SRP within the pathologic model. These observations are consistent with similar findings in a cohort of patients undergoing primary RP, where a direct relationship between more advanced pre- and postoperative tumor characteristics and persistent PSA was identified [5]. Conversely, focal therapy (OR: 0.55, p = 0.02) and a robotic approach (OR: 0.37, p < 0.001) were predictors for a lower risk to development PSA persistence after SRP. Regarding focal therapy, it can be assumed that the lower risk to develop PSA persistence after SRP results from more favorable oncologic baseline characteristics and the close follow-up of the patients, which enables recurrences to recognize earlier. However, for both (primary treatment type and surgical approach) we did not record any association with BCR (Supplementary Table 1).

It already has been assumed that persistent PSA after primary RP might result from residual PCa in the prostatic bed and/or pelvic lymphatic drainage area or occult distant metastases [9, 22]. In consequence, a similar relationship might exist between residual PCa and persistent PSA in SRP patients. Finally, these independent predictors can help to identify SRP patients at risk for PSA persistence, where it may matter in clinical decision-making.

Fourth, the current study provides BCR-free, MFS, as well as overall survival estimates in patients with persistent PSA vs. undetectable PSA after SRP. Specifically, in Kaplan Meier analyses at 84 months after SRP, BCR-free survival was 6.6 vs. 59% in patients with persistent PSA vs. undetectable PSA. Moreover, in Kaplan Meier analysis at 84 months after SRP, MFS was 71 vs. 88% for patients with persistent vs. undetectable PSA (p < 0.01). Last but not least, in Kaplan Meier analysis at 84 months after SRP, overall survival was 77 vs. 94% for patients with persistent vs. undetectable PSA (p < 0.001). In multivariable Cox regression models, persistent PSA was an independent predictor for development of BCR (HR: 5.47, p < 0.001) and for death (HR: 3.07, p < 0.01). To the best of our knowledge, we are the first to report these important observations. In consequence, the current findings of worse BCR-free and survival in patients with persistent PSA vs. undetectable PSA after SRP cannot be compared. However, in primary RP patients, a similar association between postoperatively persistent PSA and BCR was reported in multiple studies [8, 9]. Specifically, one study demonstrated, that approximately 75% of patients with persistent PSA after primary RP developed BCR [23]. Moreover, several studies demonstrated worse oncologic outcomes associated with persistent PSA after primary RP [5, 20, 24]. Specifically, in multivariable models, persistent PSA was an independent predictor for occurrence of metastases, overall mortality and cancer-specific mortality [5]. In consequence, the current study provides important findings related to overall and BCR-free survival in SRP patients. This should be acknowledged in clinical decision-making with regard to surveillance and additional treatment of these patients. However, further studies are needed in order to either corroborate or tackle our findings regarding the relationship of persistent PSA in SRP patients and their oncologic outcome.

Taken together, our observations are novel and they indicate that PSA persistence after SRP is more common than after primary RP (42 vs. 8.8%). Moreover, we provide new findings, where adverse pre- and perioperative tumor characteristics, namely higher preoperative PSA (OR 1.04), biopsy Gleason 8–10 (OR: 1.81), LNI (OR: 3.53), pathologic stage ≥T3b (OR: 1.74) as well as positive surgical margins (OR: 1.92), were independent predictors for development of PSA persistence after SRP. Finally, our observations indicate that PSA persistence after SRP, relative to undetectable PSA, is an important predictor for long-term oncologic outcome of SRP patients.

Despite these new insights, our study has some limitations. First, its retrospective nature limits the generalizability of the results. Second, PSA values could be influenced by different sensitivities of multiple PSA testing methods used in our cohort. Moreover, the time points of PSA testing were not equal. Current EAU guidelines recommend first PSA testing every six months after primary RP but there is no recommendation specifically for SRP [4]. Third, different treatment modalities of radiation therapy and focal therapy and unavailable information on the use of concomitant androgen-deprivation therapy for primary PCa treatment, might have influenced our findings. Moreover, differences in imaging after and before SRP could also have accounted for limiting homogeneity of our cohort. Specifically, PSMA-PET wasn’t available at the time of the study and could have impacted treatment in those with positive lymph node metastases or distant metastases not identified on conventional imaging. Nevertheless, the strength of this study is the large sample size of patients undergoing the generally rare procedure of SRP. In consequence, we could provide multiple novel findings that could have an impact on clinical management of patients with persistent PSA after SRP.

## Conclusions

Persistent PSA is common after SRP and represents an independent predictor for worse oncologic outcomes. Patients undergoing SRP should be closely monitored after surgery to identify those with persistent PSA. This may help identifying patients that could benefit from additional therapies.

## Supplementary information

Supplementary table 1

## Data Availability

The datasets and statistical codes generated during and/or analyzed during the current study are available from the corresponding author on reasonable request.
