# Supplementary material for: Impact of persistent PSA after salvage radical prostatectomy: a multicenter study
Source: Prostate Cancer Prostatic Dis. 2023 Oct 6;27(4):686–92. doi: 10.1038/s41391-023-00728-5 (PMC11543598; doi:10.1038/s41391-023-00728-5)
Supplement: Supplementary file 1 — Supplementary table 1 [file 41391_2023_728_MOESM1_ESM.docx]

**Supplementary table 1: Univariable Cox regression model predicting biochemical recurrence after salvage radical prostatectomy.**

|  | **Predicting BCR** | | |
| --- | --- | --- | --- |
|  | **HR** | **95%-CI** | **p -value** |
| **Undetectable PSA postoperative** | 1.00 | - | **-** |
| **Persistent PSA postoperative** | 6.12 | 4.34-8.62 | <0.001 |
| **Age** | 1.01 | 0.99-1.04 | 0.1 |
| **Preoperative PSA** | 1.01 | 1.01-1.02 | 0.02 |
| **Pathologic stage ≤T2c (referent)** | 1.00 | - | - |
| **Pathologic stage T3a** | 1.66 | 1.17-2.36 | <0.01 |
| **Pathologic stage ≥T3b** | 3.26 | 2.45-4.33 | <0.001 |
| **Pathologic Gleason ≤6 (referent)** | 1.00 | - | - |
| **Pathologic Gleason 7** | 3.41 | 1.39-8.38 | <0.01 |
| **Pathologic Gleason 8-10** | 6.82 | 2.76-16.9 | <0.001 |
| **Negative surgical margin (referent)** | 1.00 | - | - |
| **Positive surgical margin** | 1.91 | 1.48-2.45 | <0.001 |
| **Pathologic lymph node status N0 (referent)** | 1.00 | - | - |
| **Pathologic lymph node status N1** | 3.12 | 2.31-4.22 | <0.001 |
| **Pathologic lymph node status Nx** | 1.13 | 0.80-1.60 | 0.5 |
| **Percutaneous radiotherapy (referent)** | 1.00 | - | - |
| **Brachytherapy** | 1.13 | 0.83-1.55 | 0.4 |
| **Focal therapy** | 0.87 | 0.65-1.19 | 0.4 |
| **Open salvage RP (referent)** | 1.00 | - | - |
| **Robotic assisted** | 1.01 | 0.78-1.29 | 0.9 |
| **Time to salvage RP <=18 months (referent)** | 1.00 | - | - |
| **Time to salvage RP>18months** | 1.16 | 0.75-1.80 | 0.5 |

Abbreviations:; HR- Hazard Ratio; CI – Confidence interval; PSA – prostatic specific antigen; RP – radical prostatectomy.
